# Valproic Acid-Induced Hyperammonemic Encephalopathy in a Patient with Bipolar Disorder: A Case Report

**DOI:** 10.3390/brainsci10030187

**Published:** 2020-03-24

**Authors:** Meng-Yu Wu, Fang-Yu Chang, Jian-Yu Ke, Chien-Sheng Chen, Po-Chen Lin, Tzong-Shi Wang

**Affiliations:** 1 Department of Emergency Medicine, Taipei Tzu Chi Hospital, Buddhist Tzu Chi Medical Foundation, New Taipei City 231, Taiwan; skyshangrila@gmail.com (M.-Y.W.); ck4301018@gmail.com (J.-Y.K.); holeyeye@yahoo.com.tw (C.-S.C.); taipeitzuchier@gmail.com (P.-C.L.); 2 Department of Emergency Medicine, School of Medicine, Tzu Chi University, Hualien 970, Taiwan; 3 Department of Psychiatry, Taipei Tzu Chi Hospital, Buddhist Tzu Chi Medical Foundation, New Taipei City 231, Taiwan; claraczinzin@tzuchi.com.tw; 4 Department of Psychiatry, School of Medicine, Tzu Chi University, Hualien 970, Taiwan

**Keywords:** valproic acid, ammonia, hyperammonemia, hyperammonemic encephalopathy, coma

## Abstract

Valproic acid (VPA) is widely used to control various seizure disorders and psychiatric disorders. Valproic acid-induced hyperammonemic encephalopathy (VHE) is a rare but dangerous complication of VPA-induced toxicity. For this case report, several risk factors were identified, including young age, polytherapy regimens, VPA overdose, poor liver function, and carnitine deficiency. The detailed mechanisms of VHE remained unclear. Hyperammonemia may be caused by hypocarnitinemia, leading to imbalanced VPA metabolism. VHE may initially cause gastrointestinal symptoms, followed by a decreased level of consciousness and seizure. Early diagnosis of VHE is important for physicians for the timely reversal of VHE by discontinuing administration of VPA and administering lactulose or levocarnitine. Here, we describe a patient with a bipolar disorder who presented with VHE after receiving a strict vegetarian diet in our hospital. We recommend that VHE be included in the differential diagnosis of patients with high serum VPA levels and strictly vegetarian diets, especially those presenting with acute gastrointestinal symptoms.

## 1. Introduction

Valproic acid (VPA) is widely used to control various seizure disorders and psychiatric disorders, including bipolar disorder, schizophrenia, and manic psychosis. Several mechanisms of VPA’s therapeutic effects have been reported, including increasing levels of γ-aminobutyric acid (GABA) by decreasing reabsorption and catabolism, restraining neuronal repetitive firing via suppression of ion channels, and targeting the transcriptomic system to directly inhibit class I histone deacetylases (HDACs) [1,2,3,4]. In a clinical setting, patients should be monitored for effective therapeutic serum concentrations of VPA, the appropriate target range being 50–100 μg/mL [5]. However, plasma concentrations of VPA may vary in patients given the same dose of VPA. The correlation between clinical effects and plasma concentrations of VPA is poor. In some populations, VPA toxicity can occur with concentrations that are below the target range. Gastrointestinal symptoms, such as anorexia, nausea, and vomiting, are the most common early symptoms of VPA toxicity. Other adverse drug reactions include hyperammonemia, hepatotoxicity, pancreatitis, bone marrow suppression, seizure, and metabolic acidosis. VPA-induced hyperammonemia is commonly asymptomatic. It can lead to valproic acid-induced hyperammonemic encephalopathy (VHE), a rare but serious adverse effect with typical symptoms of nausea, vomiting, cognitive slowing, lethargy, coma, and seizure. Early diagnosis of VHE is important for physicians to timely reverse VHE by discontinuing administration of VPA and administering lactulose or levocarnitine. Here, we describe a rare case of acute hyperammonemic encephalopathy due to VPA in a patient with a bipolar disorder and no hepatic dysfunction. The clinical features and pathogenesis of VHE are discussed. 

## 2. Case Presentation

A 54-year-old man, with a traumatic brain injury and a 14-year history of bipolar disorder for which he was on VPA 1000 mg to 1500 mg for 14 years, presented with a 2-week history of progressive manic symptomatology. He had no past medical history of hepatitis, liver cirrhosis, type 2 diabetes mellitus, chronic renal failure, hypertension, or cardiovascular disease. On psychiatric assessment, his elated and irritable mood was accompanied by inflated self-esteem, high energy, lack of sleep, frequent shopping, rapid speech, excessive talking, and hyperactivity for 2 weeks. On physical examination, his temperature was 36.5 °C, his blood pressure was 110/69 mmHg, his heart rate was 76 beats/min, his weight was 58.9 kg, and his height was 160 cm; his Glasgow Coma Scale (GCS) score was 15 (E_4_V_5_M_6_). Initial laboratory studies revealed no abnormal results (Table 1). He was admitted to an acute psychiatry unit to control a potential bipolar disorder exacerbation with oral VPA 1400 mg per day in combination with oral quetiapine 300 mg per day.

On admission day 7, the patient complained of epigastric pain, nausea, and vomiting. On physical examination, local epigastric tenderness was noted without any muscle guarding, rebound pain, McBurney’s point tenderness, or Murphy sign. A plain abdominal film revealed no significant focal ileus or abnormal bowel gas pattern. Follow-up laboratory data revealed an elevated VPA level of 143.2 μg/mL and normal liver enzyme levels. However, on day 8, the patient lost consciousness and had a 30 s generalized tonic-clonic seizure. His GCS score was E_1_V_1_M_2_, and his pupils were 4 mm with normal light reflex bilaterally. Follow-up laboratory data revealed hyperammonemia, with an ammonia level of 488 umole/L. The patient was eventually diagnosed with VHE. VPA was discontinued on day 8, and emergency lactulose was administered to reduce his blood concentration of ammonia. In the next 6 hours, the patient’s consciousness dramatically improved, with a GCS score of E_4_V_5_M_6_. An abdominal computed tomography (CT) scan was arranged to rule out portosystemic shunt, and it revealed no abnormal findings. Hepatitis screening showed no evidence of hepatitis B or hepatitis C infection. An emergency electroencephalogram (EEG) revealed maximum intermittent delta activity at the frontal region bilaterally with symmetric, 7-Hz posterior background activity. A hyperventilation study showed no build-up response, and photic stimulation showed no photo-driving response. Moderate diffuse encephalopathy was suspected. After discontinuing treatment with VPA and administering lactulose, the patient’s gastrointestinal symptoms improved. There was no recurrent seizure or change in consciousness. Follow-up ammonia and liver enzyme levels were normal. Magnetic resonance imaging (MRI) of the brain showed punctate white matter gliosis in the frontal and parietal lobes bilaterally. A follow-up EEG revealed intermittent, diffuse slow waves in the fronto-central area bilaterally with 9- to 10-Hz alpha background waves. Moderate cortical dysfunction, including metabolic encephalopathy, was suspected. The patient regularly followed up at the outpatient clinic after discharge. A patient consent form has been obtained from the patient.

## 3. Discussion

**Figure 1:**
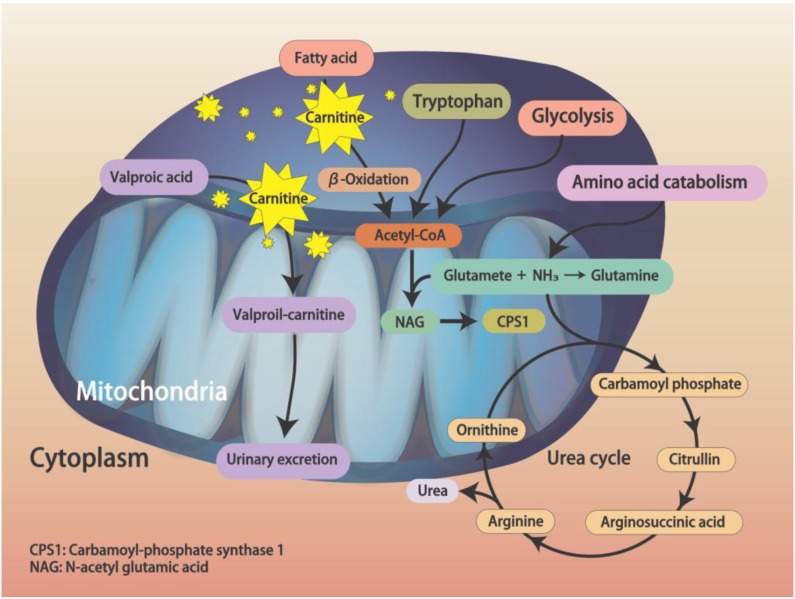
Illustration of the pathophysiological mechanisms of valproic acid metabolism and hyperammonemia in the urea cycle. Adapted from Aires et al. [8].

Hyperammonemia is a common side effect of an acute overdose or chronic use of VPA. The prevalence of VPA-induced hyperammonemia varies widely, from 16% to 100% [6]. Several mechanisms are involved in the metabolism of ammonia. Ammonia is produced via deamination of amino acids from proteins during cell metabolism [7]. In cytosol, synthesis of glutamine from glutamate and ammonia ions is catalyzed by glutamine synthetase; in mitochondria, glutaminase catalyzes the hydrolysis of glutamine to generate free ammonium ions and glutamate. Finally, ammonia is eliminated by a series of metabolic reactions in the urea cycle (Figure 1). An imbalance of ammonia catabolism may result in accumulation of intracellular and plasmatic ammonia. Timely and efficient elimination of ammonia is important due to potentially high toxicity to the nervous system. In those with a liver function deficiency or a urea cycle disorder, acute hyperammonemia may cause metabolic encephalopathy by inhibiting glutamate uptake by astrocytes; it may also induce clinical symptoms like nausea, vomiting, decreased level of consciousness, or seizure. 

At present, the detailed pathogenesis of VHE remains unclear. According to current concepts, acyl-CoA synthetase activates VPA to form valproyl-CoA in mitochondria. In the carnitine shuttle, valproylcarnitine is formed via carnitine palmitoyltransferase I and transforms into valproyl-CoA in the mitochondrial matrix. Finally, VPA is eliminated by mitochondrial β-oxidation, ω-oxidation, and urine excretion [8]. Hypocarnitinemia is a major cause of imbalanced β-oxidation and ω-oxidation and thus increased accumulation of ammonia. In hypocarnitinemia, transport of long-chain fatty acids decreases, leading to inhibited β-oxidation and acetyl-CoA production, promoting a shift to ω-oxidation, which may inhibit carbamoyl phosphate synthase I activity and decrease *N*-acetylglutamic acid (NAG) synthesis, causing accumulation of ammonia (Figure 1) [9]. In a study by Ando et al. [10], hypocarnitinemia was reported in 41% of patients. In addition, metabolism of other amino acids was involved in hyperammonemia. Serum ammonia levels were positively correlated with serum glutamate concentrations and negatively correlated with serum glutamine, citrulline, and glycine concentrations (Figure 1).

The relation between serum VPA and ammonia levels is also conflicting. In an analysis by Chicharro et al. [6], 22 of 24 studies reported an association between serum ammonia levels and serum concentrations, or doses, of VPA. However, a study by Ando, et al. [10] showed no significant correlation between serum VPA and ammonia levels. Several risk factors of VHE were identified, including age (<2 years), treatment with high serum levels of VPA (>100 mg/L) in combination with polytherapy regimens (especially phenytoin or phenobarbital), and carnitine deficiency. Carnitine deficiency may be caused by inadequate intake, enzyme deficiencies, severe liver disorders, and increased requirements for carnitine. Carnitine is produced from foods, particularly animal-based foods, via endogenous synthesis. In an analysis by Lheureux et al. [9], primary carnitine deficiency is rare and mostly genetic. Secondary carnitine deficiency is associated with various types of inborn metabolism errors and acquired medical conditions. Drug-induced carnitine deficiency is more common. In a study by Lewis et al. [11], in patients receiving chronic treatment with VPA, VPA may induce hyperammonemia by primarily inhibiting biosynthesis and depleting carnitine [9,12]. Other medicines, such as anti-HIV nucleoside analogues, ifosfamide, cisplatin, and doxorubicin, are associated with decreased carnitine levels. Further, in strictly vegetarian populations, endogenous synthesis combined with a high tubular reabsorption rate can prevent carnitine deficiency. Measuring serum carnitine levels may provide physicians with carnitine deficiency information needed to prevent VHE. 

In most patients, VPA-induced hyperammonemia does not cause encephalopathy; patients are usually asymptomatic and have normal liver function test results. In a study by Baddour et al. [13], elevated ammonia levels were not significantly associated with clinical symptoms. In addition, more clinical symptoms were noted in patients with normal ammonia levels. Lactulose and levocarnitine are common treatments for VPA-induced hyperammonemia, with success rates of 41.8% and 50.0%. The most effective treatment is VPA discontinuation, which has a success rate of 56.3%. The recovery rate with no treatment is 29.2%. Further, only 42% of untreated patients present with clinical symptoms. Based on these results, frequently checking ammonia levels in asymptomatic patients may lead to diagnostic confusion and overtreatment of hyperammonemia. However, testing ammonia levels and correcting hyperammonia are recommended for symptomatic patients undergoing VPA treatment. 

In our patient, VPA in doses of 1000 mg to 1500 mg was used for 14 years without complications. No evidence of poor liver function or hepatitis was noted. After developing a high serum VPA level due to chronic treatment with VPA, he developed gastrointestinal symptoms and then a decreased level of consciousness. The finding of hyperammonemia and his EEG findings supported the diagnosis of VHE. His encephalopathy dramatically resolved after discontinuing VPA and treating with lactulose. We suggest that VHE be included in the differential diagnosis of patients with high serum VPA levels and exhibiting gastrointestinal symptoms. We recommend that physicians keep VHE in mind.

## 4. Conclusions

Hyperammonemia may be caused by hypocarnitinemia, leading to imbalanced VPA metabolism. VPA-induced hyperammonemia does not cause encephalopathy. Patients are usually asymptomatic and have normal liver function test results. However, in symptomatic patients, VHE be included in the differential diagnosis, especially in exhibiting gastrointestinal symptoms.

## Figures and Tables

**Table 1:** The patient’s laboratory study results.

Variables	Normal Range	Patient Data				
		Day 1	Day 7	Day 8 (event)	Day 9	Day 10
White cell count	3.5–11 × 10^9^/L	8.28	8.02	7.83	---	---
Hemoglobin	12–16 g/dL	13.0	14.8	13.9	---	---
Platelet count	150–400 × 10^6^/uL	151	210	181	---	---
Blood urea nitrogen	7–18 mg/dL	8	20	15	---	---
Creatinine	0.55–1.02 mg/dL	1.0	1.0	0.9	---	---
Sodium	136–145 mmole/L	138	140	139	---	---
Potassium	3.5–5.1 mmole/L	4.0	3.7	3.7	---	---
Glucose	70–100 mg/dL	118	---	177	---	---
Alanine aminotransferase	16–63 U/L	29	19	15	---	19
Aspartate aminotransferase	15–37 U/L	27	17	10	---	15
Total cholesterol	0–200 mg/dL	149	---	---	---	---
Triglycerides	0–150 mg/dL	48	---	---	---	---
HDL cholesterol	40–60 mg/dL	41	---	---	---	---
LDL cholesterol	<130 mg/dL	89	---	---	---	---
Total bilirubin	0.0–1.0 mg/dL	---	0.44	0.31	---	---
Direct bilirubin	0.0–0.3 mg/dL	---	0.09	---	---	---
γ-glutamyltransferase	15–85 IU/L	---	21	---	---	---
Amylase	25–155 IU/L	---	44	---	---	---
Lipase	73–393 IU/L	---	148	---	---	---
Valproate, serum	50–100 μg/mL	---	143.2	124.0	57.7	---
C-reactive protein	<0.33 mg/dL	---	0.19	---	---	---
Ammonia	11–32 umole/L	---	---	488	29	26

LDL cholesterol: low density lipoprotein cholesterol; HDL cholesterol: high density lipoprotein cholesterol.
